# A novel three-dimensional system to study interactions between endothelial cells and neural cells of the developing central nervous system

**DOI:** 10.1186/1471-2202-8-3

**Published:** 2007-01-02

**Authors:** Richard Milner

**Affiliations:** 1 The Department of Pathology, University of Cambridge, Tennis Court Road, Cambridge, CB2 1QP, UK; 2 Department of Molecular and Experimental Medicine, MEM-132, The Scripps Research Institute, 10550 North Torrey Pines Road, La Jolla, CA 92037, US

## Abstract

**Background:**

During angiogenesis in the developing central nervous system (CNS), endothelial cells (EC) detach from blood vessels growing on the brain surface, and migrate into the expanding brain parenchyma. Brain angiogenesis is regulated by growth factors and extracellular matrix (ECM) proteins secreted by cells of the developing CNS. In addition, recent evidence suggests that EC play an important role in establishing the neural stem cell (NSC) niche. Therefore, two-way communication between EC and neural cells is of fundamental importance in the developing CNS. To study the interactions between brain EC and neural cells of the developing CNS, a novel three-dimensional (3-D) murine co-culture system was developed. Fluorescent-labelled brain EC were seeded onto neurospheres; floating cellular aggregates that contain NSC/neural precursor cells (NPC) and smaller numbers of differentiated cells. Using this system, brain EC attachment, survival and migration into neurospheres was evaluated and the role of integrins in mediating the early adhesive events addressed.

**Results:**

Brain EC attached, survived and migrated deep into neurospheres over a 5-day period. Neurospheres express the ECM proteins fibronectin and laminin, and brain EC adhesion to neurospheres was inhibited by RGD peptides and antibodies specific for the β1, but not the α6 integrin subunit.

**Conclusion:**

A novel 3-D co-culture system for analysing the interactions between EC and neural cells of the developing CNS is presented. This system could be used to investigate the reciprocal influence of EC and NSC/NPC; to examine how NSC/NPC influence cerebral angiogenesis, and conversely, to examine how EC regulate the maintenance and differentiation of NSC/NPC. Using this system it is demonstrated that EC attachment to neurospheres is mediated by the fibronectin receptor, α5β1 integrin.

## Background

During development the CNS is vascularized by angiogenesis, in which blood vessels covering the leptomeningeal surface of the brain turn and grow in towards the CNS parenchyma [1-3]. EC detach from the growing blood vessels, to proliferate and migrate deeper into the cerebral parenchyma. Evidence suggests that the patterns of cerebral angiogenesis are closely matched to the requirements of the surrounding neural tissue. This is well illustrated in the developing cerebral cortex in which emergence of the different neuronal layers is closely followed by the appearance of new blood vessels within each layer [2,4]. This process proceeds in a timely and well-coordinated manner. Studies show that cells of the developing CNS regulate angiogenesis by secretion of vascular endothelial growth factor (VEGF) [5,6], and by production of fibronectin along the pathway of migrating endothelial cells [7]. More recently, it has been shown that NSC promote vascular tube formation in brain EC in vitro, by a nitric oxide-mediated mechanism [8].

Conversely, recent studies have highlighted the role of blood vessels in maintaining the stem cell niche within the CNS [3,9]. Therefore, communication between EC and neural cells of the developing CNS appears to be a two-way street, with each cell type influencing the behaviour of the other. Consequently, a better understanding of the molecular signals that mediate these interactions may be important not only for angiogenesis, but also for the maintenance and differentiation of NSC/NPC within the CNS.

Many factors have been implicated in angiogenesis, including the growth factors VEGF and bFGF, the ephrins, the angiopoietins [10-12], and molecules of the ECM, such as fibronectin and laminin [13,14]. Interestingly, many of these factors also influence NSC/NPC behaviour [15], raising the notion that NSC/NPC and EC are both regulated by an evolutionary-conserved set of molecular signals, that help to co-ordinate the establishment and maintenance of the developing CNS.

The ECM provides signals that promote EC survival, proliferation, migration and differentiation [16-20]. The major class of ECM receptors are the integrins, which are expressed at the cell surface as noncovalently linked αβ heterodimers [21-24]. Within the CNS, β1 integrins are expressed at high levels by EC [25-28], and their expression is tightly regulated in different pathological states including stroke, multiple sclerosis and neoplasia [26,29-32]. An important role for β1 integrins in cerebral angiogenesis was suggested by the recent study from this laboratory describing β1 integrin expression during postnatal CNS development [27]. This revealed that brain EC showed a developmental switch in their expression of β1 integrins, from predominantly fibronectin receptors (α4β1 and α5β1 integrins) in the early stages of angiogenesis to laminin receptors (α1β1 and α6β1 integrins) in the adult stable state. This suggests that the switch from fibronectin to laminin signalling might be an important factor regulating EC behaviour, switching the cells from an angiogenic phenotype into a more stable differentiated state.

In order to test this idea we recently examined the influence of different ECM proteins on brain EC behaviour in vitro. This showed that fibronectin was the most effective protein at promoting EC survival and proliferation, and that this effect was mediated via the α5β1 and αvβ3 integrins by a MAP kinase-dependent signalling pathway [33]. This study, like many others, was performed on simple two-dimensional (2-D) substrates. In light of the increasing realization that cells within 3-D systems show different modes of behaviour than those in 2-D [34,35], the aim of the current study was to develop a 3-D system to examine interactions between EC and cells of the developing CNS. Fluorescent-labelled brain EC were seeded onto neurospheres, free-floating spheres of cells containing predominantly NSC, NPC and smaller numbers of differentiated cells. This system models the outside-in EC invasion into the brain that occurs during development [1-3]. In this system, the potential role of β1 integrins in mediating EC attachment and migration into neurospheres has been examined.

## Results

### Brain EC attach to neurospheres

The aim of this study was to develop a 3-D system to examine interactions between EC and cells of the developing CNS. The neurosphere system was utilized, in which postnatal neural cells are cultured under non-adherent conditions in the presence of the growth factors EGF and FGF2. In these cultures, NSC proliferate to form free-floating neurospheres, containing predominantly NSC, NPC and smaller numbers of differentiated cells of neuronal or glial lineage [36-38]. As an initial step in this study, primary cultures of mouse brain EC were seeded onto the surface of mouse neurospheres to determine whether EC would attach and migrate into neurospheres, as a means of recapitulating the EC ouside-in invasion of the CNS parenchyma that occurs during development.

**Figure 1:**
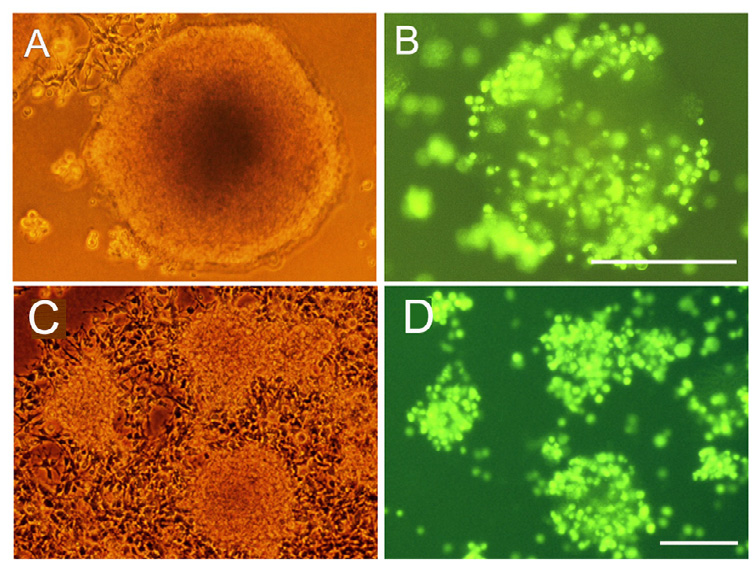
Brain EC attachment to neurospheres. Mouse brain EC were prepared, labelled with the fluorescent cell tracker marker (green), plated onto mouse neurospheres, and then cultured under serum-free conditions, as described in Methods. After 18 hours (A and B) and 5 days (C and D), EC adhesion to neurospheres was examined by fluorescent microscopy. Representative cultures are shown by phase (A and C) and fluorescent (B and D) images. Scale bars = 100 μm. Note that after 18 hours co-culture many fluorescent-labelled EC had attached to neurospheres and that after 5 days, many EC were still present, and were concentrated on or within neurospheres.

First, mouse neurospheres were plated in a small volume (25 μl) of neurosphere media in the centre of wells within a 24-well plate. Each drop contained between 20–25 neurospheres of size range 80–200 μm. Neurospheres were used for the first 4 passages and then discarded. After leaving the neurospheres 15 minutes to settle down on the plastic base, a 10 μl single cell suspension containing 20,000 cell-tracker-labelled mouse brain EC was suspended over the neurospheres. The small volumes were designed to maximise interactions between EC and neurospheres. After 2 hours incubation, 0.5 ml of neurosphere media was gently added to the well, and the plates returned to the incubator. EC adhesion to neurospheres was examined after 18 hours co-culture, by observing the living cultures under fluorescent microscopy. As shown in Figure 1 (panels A and B), after 18 hours incubation, it appeared that most neurospheres were covered in fluorescent-labelled EC. To assess the proportion of EC that had attached to neurospheres, the number of attached EC was first counted using fluorescent microscopy. However, this proved very difficult because of the 3-D nature of the culture system and the mobile nature of free-rolling neurospheres. Therefore EC attachment to neurospheres was assessed in an alternative approach by quantifying the number EC that did not attach to neurospheres. This was achieved by first, removing all neurospheres and EC from the non-adherent plates into a centrifuge tube and allowing the neurospheres to separate out by gravity. Next, the number of EC in the remaining supernatant was counted using a haemocytometer. This showed that of the 20,000 EC added to the neurosphere cultures, 63.2 ± 14.3% had not attached to a neurosphere (n = 6 experiments). In an additional approach the number of fluorescent-labelled EC that attached to neurospheres was also quantified by flow cytometry. In this approach, the sedimented neurospheres containing attached EC were dissociated with trypsin into a single cell suspension before analysis by flow cytometry to quantify the absolute number of fluorescent-labelled EC present within the cell population. This showed that of the original 20,000 EC plated onto the neurospheres 30.8 ± 5.6% (n = 3 experiments) had attached to a neurosphere. These results are largely consistent and show that approximately one-third of the originally-plated EC attach to the neurospheres under the conditions in this assay system.

The co-cultures were maintained for a further 5 days to assess whether the attached EC would survive for longer periods. This was important to establish because the EC-neurosphere co-cultures were maintained in serum-free media, so as to prevent NSC/NPC adhesion and differentiation, and EC do not normally survive in serum-free conditions [39,40]. However, as illustrated in Figure 1 (panels C and D), after 5 days culture, many EC still remained attached to neurospheres, showing that in this system, EC do survive in the absence of serum. This suggests that EC in contact with neurospheres obtain sufficient pro-survival signals, most likely as a result of their strong adhesion to neurospheres, and from pro-survival factors released from the NSC/NPC within the neurospheres.

### Brain EC migrate into neurospheres

**Figure 2:**
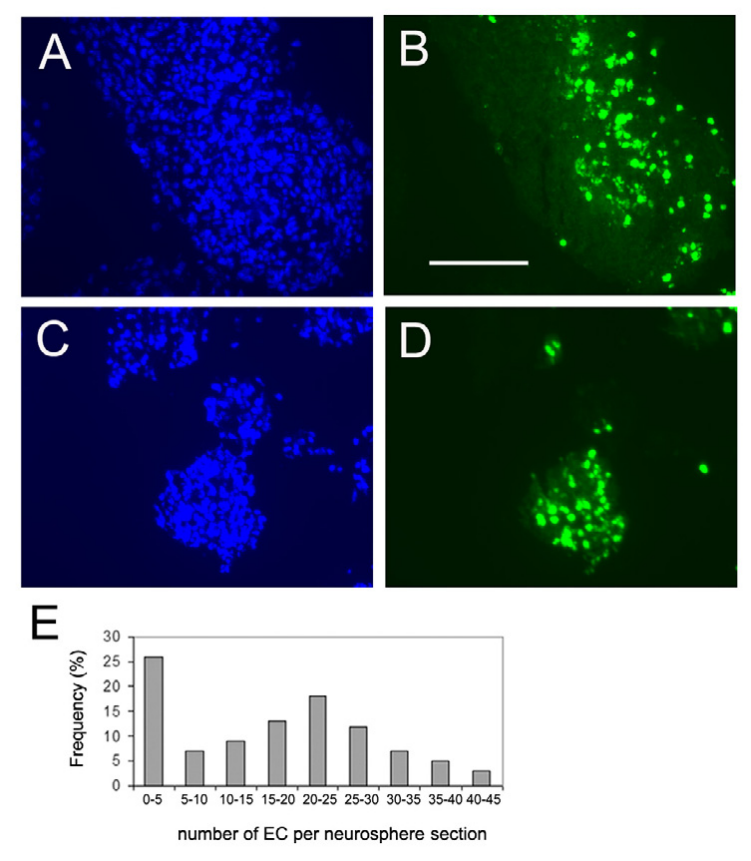
Brain EC migration into neurospheres. Mouse brain EC were prepared, labelled with cell tracker, and then plated onto mouse neurospheres, as described in Methods. 5 days later, the distribution of fluorescent-labelled EC within neurospheres was examined by fluorescent microscopy of frozen sections of EC-neurosphere co-cultures, using Hoechst (blue) to identify all cell nuclei (A and C) and the FITC channel (green) to identify the cell tracker-labelled EC (B and D). Scale bar = 100 μm. Panel E shows a histogram of the distribution of EC number within neurospheres in a representative experiment (n = 3). Note that EC were detected in the majority of neurospheres.

Having established that EC can attach to neurospheres and survive for periods of up to 5 days, the next aim was to determine whether EC actively migrated into neurospheres, or just remained superficially attached to the neurosphere surface. To examine this, fresh frozen sections of 5 day-old EC-neurosphere co-cultures were analysed for the distribution of fluorescent-labelled EC within the neurospheres. As shown in Figure 2, many fluorescent-labelled EC were detected within neurospheres, and these cells were distributed not just at the edge of the neurospheres, but throughout the entire neurosphere structure. To examine how effectively EC migrate into neurospheres, the number of EC within neurospheres was determined by counting the number of fluoresecent-labelled EC in 10 different 10 μm sections of the EC-neurosphere co-cultures, each containing between 10–15 neurospheres. As shown in Figure 2E, this showed that the majority of neurospheres contained EC, though approximately 25% of neurospheres contained no EC. The observation that some neurospheres contained absolutely no EC, even at the neurosphere surface, supports the idea that EC failed to contact these neurospheres in the original plating procedure. To gain some idea of the survival rate of EC within this co-culture system, a calculation was performed based on the cell counts of EC within co-culture sections. In light of the observation that within this system the average neurosphere diameter was 150 μm, it was estimated that by counting EC in 15 sections of 10 μm each, this would include all EC present. Using this calculation, it was determined that over the course of three separate experiments, that of the 20,000 EC originally co-cultured with neurospheres, 20.55 ± 7.8% EC survived up to day 5 of the experiment. In light of the observation that neurospheres are capable of fusing in culture [41], it is possible that the appearance of EC within neurospheres could be explained by neurosphere fusion and incorporation of the surface-attached EC into the centre of aggregated neurospheres. To exclude the possibility that this might be the reason for detecting EC within neurospheres, single neurospheres were plated into the centre of wells within 24-well tissue culture plates and then seeded with fluorescent-labelled EC. Subsequent analysis of frozen sections by fluorescent microscopy revealed that fluorescent-labelled EC were detected throughout the neurospheres plated as single neurospheres, confirming that EC migrate readily through neurospheres.

**Figure 3:**
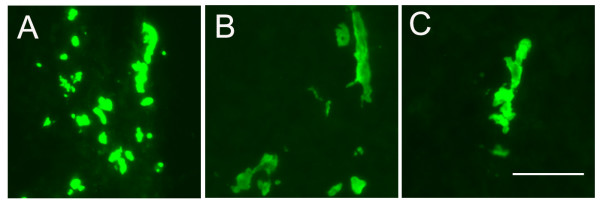
The morphology of brain EC in neurospheres after 5 days. Mouse brain EC were prepared, labelled with cell tracker, and plated onto mouse neurospheres, as described in Methods. 5 days later, brain EC morphology was analysed in frozen sections under fluorescent microscopy. A-C are representative examples of the morphologies observed. Scale bar = 50 μm. Note that after 5 days co-culture, some brain EC form linear structures within neurospheres, reminiscent of the early vascular structures observed in the developing CNS.

**Figure 4:**
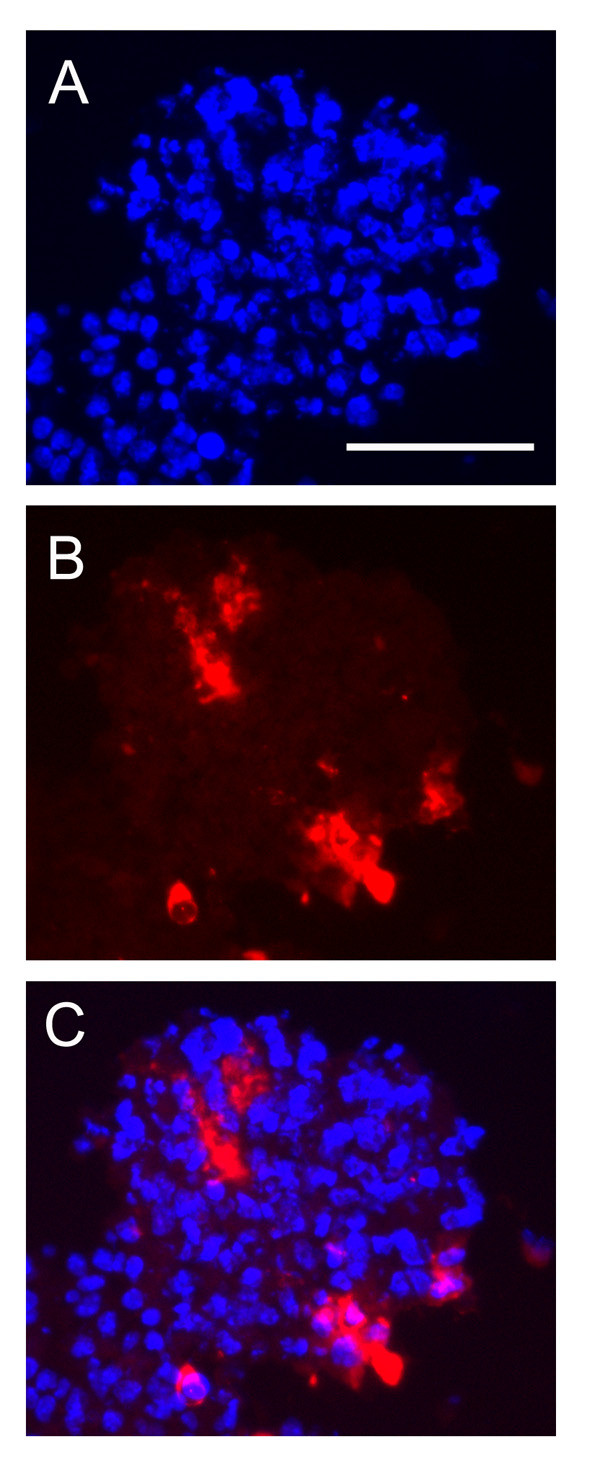
CD31 immunofluorescence of EC-neurosphere co-cultures after 5 days culture. Mouse brain EC were plated onto mouse neurospheres, as described in Methods. Five days later, CD31 immunohistochemistry was performed on frozen sections using Hoechst to label all cell nuclei (A) and CD31 (Cy-3 conjugated secondary antibody, (B) to identify EC. Merged channels (C). Scale bar = 50 μm. Note that after 5 days co-culture, CD31-positive structures were observed that resembled early vascular structures.

When the morphology of the fluorescent-labelled EC inside neurospheres was examined (Figure 3), it was noticed that EC were often grouped together in small aggregates, and in a small number of cases, the EC had formed linear groups of cells, reminiscent of those observed during the early stages of vascular development in the CNS [3,27]. These linear groups of EC were never found in the early stages of EC-neurosphere co-culture, and only became apparent after 5 days culture. Because some studies have reported that NSC within neurospheres can differentiate into EC [42], next, expression of the endothelial-specific marker CD31 (PECAM-1) was examined by immunofluorescence. This showed that cells within neurospheres not containing any fluoresecent-labelled EC did not express CD31. However in neurospheres receiving EC, CD31-positive cells were distributed throughout the neurospheres, and in some cases the CD31-positive cells showed a linear vascular-like appearance (Figure 4). Furthermore, CD31-positive cells negative for the fluorescent cell-tracker label were never detected, suggesting that NSC/NPC within the neurosphere did not differentiate into EC.

### Neurospheres express the ECM proteins fibronectin and laminin

**Figure 5:**
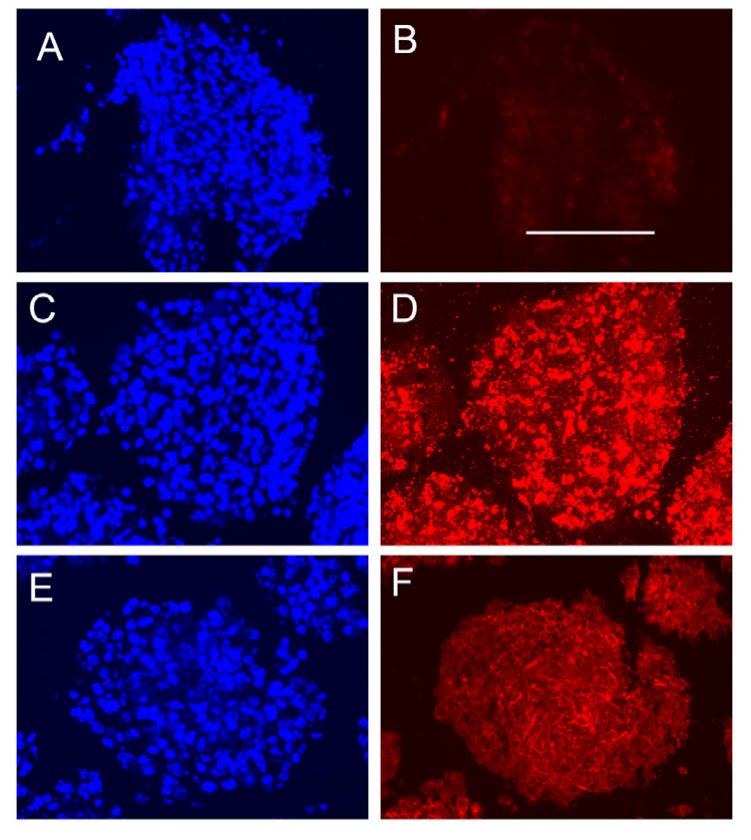
Expression of fibronectin and laminin within neurospheres. Frozen sections of mouse neurospheres were prepared as described in Methods and expression of fibronectin and laminin examined by immunofluorsecent microscopy. Neurospheres were labelled with normal rabbit serum (B), or primary antibodies against fibronectin (D) or laminin (F), before labelling with a Cy-3 conjugated secondary anti-rabbit antibody. All cell nuclei of the neurospheres were identified by Hoechst labelling (A, C, E). Scale bar = 100 μm. Note that fibronectin was expressed throughout the neurosphere, with a pattern that outlined individual cell bodies, while laminin labelled cell processes between cells, and was expressed predominantly by cells nearer the centre of the neurosphere.

A previous report from this laboratory demonstrated that brain EC express integrin receptors for fibronectin and laminin [33]. To address whether these two ECM proteins are expressed within neurospheres, immunohistochemistry was performed on frozen neurospheres, using polyclonal antibodies specific for fibronectin and laminin. This showed that both ECM ligands were expressed within neurospheres (Figure 5). Fibronectin was expressed throughout neurospheres, with a pattern that outlined individual cell bodies of the neurosphere. Laminin was expressed in an intricate fibrillar pattern, outlining cell processes between cells. Interestingly, while fibronectin expression was distributed throughout the neurosphere structure, laminin expression was highest on cells within the core of neurospheres.

### Integrins promote EC attachment to neurospheres

This study has demonstrated that primary mouse brain EC attach, migrate and survive within mouse neurospheres, thus validating this system as a tool to investigate the molecular mechanisms mediating interactions between EC and NSC/NPC within the developing CNS. The specific focus of this research group is to understand the role of integrins in regulating EC behaviour within the CNS. Previously our studies demonstrated a developmental switch in EC integrins, from fibronectin-mediated signalling during the early stages of angiogenesis, to laminin-mediated signalling during endothelial differentiation [27]. To extend this work, the next set of studies were designed to test the hypothesis that β1 integrins provide important instructional cues that drive the early events of angiogenesis in the CNS, such as EC adhesion and migration into the developing CNS.

**Figure 6:**
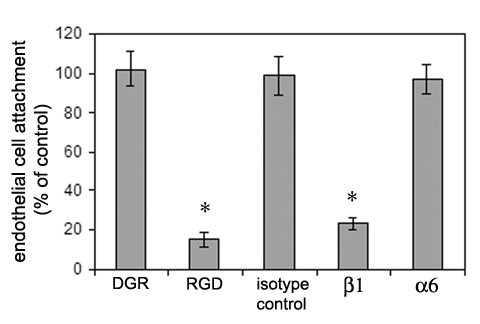
Analysis of the roles of specific integrins in mediating brain EC attachment to neurospheres. Cell-tracker-labelled mouse brain EC were plated onto mouse neurospheres, in the presence of the different integrin function-blocking reagents: RGD peptides, the β1 integrin monoclonal antibody Ha2/5, or the α6 integrin monoclonal antibody GoH3. Control DGR peptides, or an isotype-matched monoclonal antibody were included for controls. 18 hours later, EC adhesion to neurospheres was quantified. Adhesion is expressed as the percentage of EC adhesion to neurospheres under control conditions (no reagents added); all points represent the mean ± SD of four experiments. * indicates statistically significant effect (p < 0.01). Note that EC adhesion to neurospheres was inhibited by RGD peptides and the β1 integrin monoclonal antibody, but not by the α6 integrin antibody.

To investigate the importance of integrins in mediating adhesive interactions between EC and neurospheres, the impact of integrin function-blocking reagents on EC attachment to neurospheres was evaluated. Fluorescent-labelled EC were added to cultures of neurospheres in the presence of the different function-blocking reagents: control DGR peptide, blocking peptide RGD (which targets the α5β1 integrin but not α6β1), isotype control antibody, anti-β1 integrin antibody (Ha2/5), or the anti-α6 integrin antibody, GoH3. As before, 10 μl containing 20,000 fluoresecent-labelled EC was suspended over a 25 μl drop containing 20–25 neurospheres of size range 80–200 μm. After 18 hours culture the effect of integrin blockade on EC adhesion to neurospheres was quantified, by first, measuring the proportion of EC that remained unattached to NS and second, by flow cytometry of the neurosphere population to quantify the number of fluoresecent-labelled EC that had attached to neurospheres. As shown in Figure 6, when compared to control conditions, EC attachment to neurospheres was significantly inhibited both by the RGD peptides (reduced to 15.4 ± 3.5% of the control value, p < 0.01) and by the pan-β1 integrin antibody (reduced to 23.4 ± 3.3% of the control value, p < 0.01), but not by the control DGR peptides, isotype-control antibody or by the anti-α6 antibody. Flow cytometric analysis yielded very similar results (not shown). Taken together, these results suggest that EC attachment to neurospheres is dependent on the fibronectin-binding α5β1 integrin but not the laminin-binding α6β1 integrin.

## Discussion

In this study a novel 3-D co-culture system to study the interactions between brain EC and neural cells of the developing CNS is presented. Using neurospheres as a model of the developing CNS, brain EC were seeded onto neurospheres in an attempt to mimic the outside-in EC invasion into the CNS that occurs during development [1-3]. The studies demonstrate that EC attach, migrate and survive inside neurospheres. In addition, to investigate the potential role of integrins in promoting the early stages of cerebral angiogenesis, the studies revealed that neurospheres express fibronectin and laminin, the appropriate ECM ligands for EC β1 integrins, and that EC attachment to neurospheres is mediated by the α5β1 integrin.

Angiogenesis has become intensely studied because of its involvement in many pathological processes, particularly neoplasia [12,43,44]. In the CNS, angiogenesis is an important part of normal development [1,2,4], but it also occurs in a number of pathological conditions in the adult CNS, including arteriovenous malformations (AVMs) [45,46], neoplastic conditions [47-51], and cerebral ischaemia [52-54]. Therefore an improved understanding of the molecular events that underlie brain angiogenesis is of high priority. The growing realization that cells behave differently in 2-D versus 3-D environments [34,35,55] has led many to believe that it may be more informative to study EC behaviour in 3-D rather than 2-D systems. To address this, many studies have used matrigel, fibrin or collagen 3-D gels to try and create a more complex 3-D environment [55-59]. However the drawback of these approaches is that while they are 3-D, they may be too simplistic, containing only one or two ECM proteins, and do not contain other cell types that may be important for orchestrating angiogenesis during development. The novel 3-D co-culture system described in this study may be a significant step forward for several reasons. First, the neurosphere system provides a simple 3-D model of the developing CNS, containing the major cell types present within the developing CNS at the time of angiogenesis, including NSC, NPC and other more differentiated cells [36-38]. Second, unlike culture systems that use postnatal astrocytes [60-62], the majority of neural cells within the neurosphere are at an immature stage of development, thus closely mimicking the environmental cues that migrating EC will encounter as they start to invade the developing CNS [36-38]. Third, unlike the complexity of the developing in vivo situation, the EC-neurosphere co-culture model is relatively easy to establish, and is amenable to manipulation and analysis.

This 3-D system could be used to address several different fundamental questions relating to interactions between EC and NSC/NPC. First, it could be used to investigate the influence of EC on NSC/NPC maintenance, proliferation and differentiation. In light of the notion that blood vessels may be an important component of the stem cell niche [9], and the findings by Temple et al [3], that EC promote NSC proliferation and induce NSC differentiation along the neuronal pathway, it will be interesting to investigate this influence in the 3-D system presented here. Second, it could be used to investigate the influence of NSC/NPC on EC behaviour and thus angiogenesis. Previous data shows that neurons, astrocytes and NSC produce soluble factors that promote angiogenesis [5,6,8]. The system presented here would be a valuable tool for characterising NSC/NPC factors involved in this regulation. Third, it provides a good system to examine the role of specific candidate molecules in regulating the different stages of brain angiogenesis. In the work presented here, it is demonstrated that the fibronectin-α5β1 integrin interaction is important for the initial EC adhesion to neurospheres. In future studies this avenue will be extended by using EC derived from integrin null mice and siRNA to examine the role of individual integrins and key components of the integrin-associated intracellular signalling pathways. In this initial study it was noted that while EC attach and migrate into the body of neurospheres, the actual degree of vascular tube formation after 5 days co-culture under baseline conditions was relatively small. In future work, the ability of well-described angiogenic factors such as bFGF [12,63] or TGF-β1 [64,65] to promote vascular tube formation will be evaluated in this system.

This 3-D system was used to explore the role of ECM-integrin interactions in regulating some of the early events mediating brain angiogenesis. Importantly, it was found that EC attached rapidly and migrated into the centre of neurospheres. Previously we have shown that in the developing CNS, EC switch from a predominantly fibronectin signalling pathway (high expression of α4 and α5 integrins and surrounded by fibronectin) to a laminin signalling pathways (high expression of α1 and α6 integrins and surrounded by laminin) [27]. In keeping with the observations of others [66] the work presented here confirms that both fibronectin and laminin are expressed by neural cells within the neurosphere, thus providing the ECM ligands for EC integrins to interact with. Interestingly, while fibronectin was expressed throughout the neurospheres, laminin expression was focussed preferentially at the centre of neurospheres. In addition, function-blocking studies showed that the α5β1 integrin, a fibronectin receptor, is important in promoting EC attachment to neurospheres. This is consistent with the observation that EC migrating from the brain surface into the parenchyma during development are in close contact with a fibronectin-rich matrix [1], and also from our previous work showing that fibronectin-mediated interactions are dominant in the early stages of cerebral angiogenesis [27]. Taken together, these findings suggest that fibronectin-mediated events play an important role in the early stages of EC adhesion and migration in the developing CNS.

## Conclusion

A novel 3-D system for investigating interactions between EC and NSC/NPC is presented. When plated onto neurospheres, brain EC attached, survived and migrated into neurospheres. This system could be used to investigate the reciprocal influence of EC and NSC/NPC; to examine how NSC/NPC influence cerebral angiogenesis, and conversely, to examine how EC regulate the maintenance and differentiation of NSC/NPC. Here, this system has been used to show that EC attachment to neurospheres is mediated by the fibronectin receptor, α5β1 integrin.

## Methods

### Cell culture

All cell cultures were maintained in a humidified incubator at 37°C and 5% CO_2_. Primary cultures of mouse brain EC were prepared as previously described [33] according to the method of Sapatino [67], with some modifications. Briefly, brains of adult C57BL6 mice (2–3 months old) were removed, cleaned of meninges and external blood vessels, then finely chopped, and dissociated for one hour in an enzymatic solution containing 30 U/ml papain (Worthington Biochemicals, Lorne Laboratories, United Kingdom), 0.24 mg/ml L-cysteine (Sigma) and 40 μg/ml DNAse I type IV (Sigma) in 1 ml MEM-HEPES, as previously described for mixed glial cultures [68]. After incubation, the disrupted brain tissue was triturated 10 times with a 10 ml syringe, first through a 19-gauge needle, then through a 21-gauge needle. The brain tissue was then added to a universal 25 ml tube containing 22% bovine serum albumin (BSA), and centrifuged at 1000 g for 10 minutes, in order to separate out the myelin that is retarded at the top of the tube, from the vascular tubes and other cells at the bottom of the tube. This process was repeated 2 times, each time re-centrifuging the myelin fraction to harvest more cells and tubes. Finally, the collected vascular tubes and cells were resuspended and filtered through a 40 μm cell strainer (Falcon, Oxford, United Kingdom) to separate the vascular tubes from single cells. The tubes were then washed and centrifuged in MEM-HEPES before being resuspended in endothelial cell growth media (ECGM) consisting of Hams F12, supplemented with 10% FCS, Heparin, ascorbic acid, L-glutamine (all from Sigma) and endothelial cell growth supplement (ECGS) (First Link, Birmingham, United Kingdom). T25 tissue culture flasks (Falcon) had previously been coated for 2 hours with collagen type I (Sigma), which was then cross-linked in a box containing ammonia vapour for 30 minutes before being rinsed 2 times with PBS and left in the incubator for 1 hour to equilibrate. The brain EC were then added to the collagen-coated flasks and left to grow at 37°C and 5% CO_2_. The EC purity of the cultures was assessed by live immunostaining with antibodies for the EC surface markers CD31 (PECAM-1) and CD105 (endoglin), and determined as greater than 95% pure EC. In these experiments EC were used only for the first two passages and then discarded.

Primary cultures of neurospheres were obtained from postnatal (P0–P2) C57BL6 mouse brains as described previously [69]. Briefly, spheres of neural precursors were grown in uncoated Iwaki non-adherent T25 tissue culture flasks (Bibby Sterilin, Stone, United Kingdom) in DMEM/Hams F12 (Sigma, Poole, United Kingdom) (50:50) supplemented with 1% B27 (Life Technologies, Paisley, United Kingdom), 20 μg/ml epidermal growth factor (EGF, Calbiochem, Nottingham, United Kingdom) and 20 μg/ml fibroblast growth factor-2 (FGF2, Peprotech, London, United Kingdom). Cells were plated at an initial plating density of two brains per T25 flask. After 3 days the contents of each T25 flask were centrifuged at 800 rpm for 3 minutes and the neurosphere pellet resuspended through a 1 ml pipette and plated into four fresh T25 flasks. Neurospheres were subsequently passaged every 5–7 days into fresh flasks and fresh media.

### Antibodies and peptides

The following antibodies used in immunocytochemistry, immunohistochemistry and function-blocking studies were obtained from: (1) Pharmingen (La Jolla, CA): monoclonal antibodies against CD31 (PECAM-1, clone MEC13.3), CD105 (endoglin, clone MJ7/18), α6 integrin (clone GoH3), β1 integrin (clone Ha2/5), (2) Sigma: rabbit polyclonal antibodies against fibronectin and laminin. The FITC and Cy-3-conjugated secondary antibodies were obtained from DAKO (Ely, United Kingdom). The GRGDS (function-blocking) and SDGRG (control-inactive) peptides were obtained from Sigma.

### Immunocytochemistry

For assessment of EC purity, cells were cultured on collagen I or fibronectin (both from Sigma) coated glass coverslips in ECGM. Cells were blocked in 5% normal goat serum (NGS) in PBS for 30 minutes before being live-labelled with monoclonal antibodies specific for CD31 or CD105 for 1 hour at room temperature. Cells were then washed in PBS and incubated with secondary FITC-conjugated secondary antibodies for 1 hour at room temperature and washed again. The cells were then fixed in acid/alcohol (95:5) at -20°C for 30 minutes, before being extensively washed and incubated with Hoechst stain (Sigma) for 10 minutes at room temperature to label all cell nuclei. Coverslips were then washed again before being mounted in aquamount (Polysciences, Warrington, PA). Fresh frozen 10 μm sections of neurospheres and neurospheres seeded with EC were prepared by rapid freezing of neurospheres in OCT, and cryostat sections prepared at -20°C. The sections were dried and then fixed briefly in acetone/methanol (50:50) for 60 seconds before being rinsed extensively in PBS and blocked for 30 minutes at room temperature in 5% NGS in PBS. Sections were then immunostained with primary antibodies against fibronectin or laminin (1:100 dilution) for 1 hour at room temperature, washed for 10 minutes in PBS, incubated for 1 hour with secondary antibodies (1:200 dilution), washed for 10 minutes, and then cell nuclei labelled with the Hoechst stain for 10 minutes. Finally sections were washed in PBS and mounted in aquamount.

### Analysis of endothelial cell interactions with neurospheres

Mouse neurospheres were plated in a small volume (25 μl) of neurosphere media in the centre of wells within a 24-well plate. Each drop contained between 20–25 neurospheres of size range 80–200 μm. Neurospheres were used for the first 4 passages and then discarded. After leaving the neurospheres 15 minutes to settle down on the plastic base, a 10 μl single cell suspension containing approximately 20,000 cell-tracker-labelled brain EC was suspended over the neurospheres. The small volumes were designed to maximise interactions between EC and neurospheres. After 2 hours incubation, 0.5 ml of neurosphere media was gently added to the well, and the plates returned to the incubator. Primary cultures of mouse brain EC were labelled with cell tracker (Molecular Probes, Eugene, OR, USA) according to the manufacturers instructions. Briefly, ECGM was removed and EC incubated with 1:1000 dilution of cell tracker in serum free Hams F12 media for 45 minutes. The labelling media was then removed and EC incubated in ECGM for a further 1 hour before being trypsinised off the dish. Trypsin (Life Technologies) was blocked with DMEM containing 10% FCS and the EC pelleted at 1200 rpm. Labelled EC were resuspended in neurosphere growth media at a cell density of 2 × 10^6 ^EC/ml, and 10 μl volumes of this cell suspension added gently on top of the previously prepared neurospheres. The cultures were then left to incubate for 2 hours before adding another 0.5 ml neurosphere media to each well and the cultures returned to the incubator. These cultures were left undisturbed for 18 hours and then examined under the fluorescent microscope for evidence of fluorescent-labelled EC attachment to neurospheres.

EC attachment to neurospheres was quantified by the reverse approach of counting the number of EC that did not attach to neurospheres. Briefly, neurospheres and EC were removed from the non-adherent plates into a centrifuge tube and left for 2 minutes to allow the neurospheres to settle down by gravity. The residual supernatant containing unattached EC was centrifuged at 1500 rpm to pellet the EC, and then the number of EC measured using a haemocytometer. EC attachment to neurospheres was also quantified by flow cytometry of trypsin-dissociated EC-neurosphere co-cultures. Neurospheres were allowed to settle by gravity for 2 minutes and then dissociated in 0.05% trypsin-EDTA (Sigma) for 5 minutes at 37°C. The number of fluoresecent-labelled EC within the co-culture was then quantified by flow cytometry of the mixed cell population and calculated as a percentage of the number of EC initially plated. In the function-blocking experiments, the blocking reagents were added to the EC for 15 minutes before plating on neurospheres, and then maintained for the duration of the experiment. GRGDS (RGD) and SDGRG (DGR) peptides were included at 0.1 mg/ml, and the β1 (Ha2/5) and α6 (GoH3) integrin antibodies included at 10 μg/ml. Each condition was repeated in duplicate in each experiment. The results are expressed as the mean ± SD of four experiments. Statistical significance was assessed by using the Student's paired t test in which P < 0.05 was defined as statistically significant.

For the analysis of EC incorporation into neurospheres, frozen sections of EC-neurosphere co-cultures were dried and then fixed briefly in acetone/methanol (50:50) for 60 seconds before being rinsed in PBS and mounted in aquamount. To determine the proportion of neurospheres that contain EC, the number of fluorescent-labelled EC within neurospheres was measured in 10 different 10 μm sections of the EC-neurosphere co-cultures, each section containing between 10–15 neurospheres. To calculate the approximate survival rate of EC within this co-culture system, we performed a calculation based on our cell counts of EC within the co-culture sections. As the average neurosphere diameter is 150 μm, we reasoned that by counting the EC in 15 sections of 10 μm each, this would include all EC present. The number of fluorescent-labelled EC within neurospheres was counted within 3 separate sections, and this number multiplied by a factor of 5 to give an estimate of the total number of EC present within the co-culture.
